# Improving access to health care for malaria in Africa: a review of literature on what attracts patients

**DOI:** 10.1186/PREACCEPT-2317562776368437

**Published:** 2012-02-23

**Authors:** James Kizito, Miriam Kayendeke, Christine Nabirye, Sarah G Staedke, Clare IR Chandler

**Affiliations:** 1 Uganda Malaria Surveillance Project, Infectious Diseases Research Collaboration, Kampala, Uganda; 2 Department of Clinical Research, London School of Hygiene and Tropical Medicine, London, UK; 3 Department of Global Health & Development, London School of Hygiene and Tropical Medicine, London, UK

**Keywords:** Access, Treatment seeking, Malaria, Attracting factors, Health care providers

## Abstract

**Background:**

Increasing access to health care services is considered central to improving the health of populations. Existing reviews to understand factors affecting access to health care have focused on attributes of patients and their communities that act as 'barriers' to access, such as education level, financial and cultural factors. This review addresses the need to learn about provider characteristics that encourage patients to attend their health services.

**Methods:**

This literature review aims to describe research that has identified characteristics that clients are looking for in the providers they approach for their health care needs, specifically for malaria in Africa. Keywords of 'malaria' and 'treatment seek*' or 'health seek*' and 'Africa' were searched for in the following databases: Web of Science, IBSS and Medline. Reviews of each paper were undertaken by two members of the team. Factors attracting patients according to each paper were listed and the strength of evidence was assessed by evaluating the methods used and the richness of descriptions of findings.

**Results:**

A total of 97 papers fulfilled the inclusion criteria and were included in the review. The review of these papers identified several characteristics that were reported to attract patients to providers of all types, including lower cost of services, close proximity to patients, positive manner of providers, medicines that patients believe will cure them, and timeliness of services. Additional categories of factors were noted to attract patients to either higher or lower-level providers. The strength of evidence reviewed varied, with limitations observed in the use of methods utilizing pre-defined questions and the uncritical use of concepts such as 'quality', 'costs' and 'access'. Although most papers (90%) were published since the year 2000, most categories of attributes had been described in earlier papers.

**Conclusion:**

This paper argues that improving access to services requires attention to factors that will attract patients, and recommends that public services are improved in the specific aspects identified in this review. It also argues that research into access should expand its lens to consider provider characteristics more broadly, especially using methods that enable open responses. Access must be reconceptualized beyond the notion of barriers to consider attributes of attraction if patients are to receive quality care quickly.

## Background

Increasing access to health care services is considered central to improving the health of populations. Prompt access to malaria diagnosis and treatment is a key component of the Roll Back Malaria Partnership's Global Malaria Action Plan to reduce deaths attributable to malaria to near-zero by 2015 [1]. Much research has been conducted to understand how to improve access to services in low-resource settings, including for malaria care, and frameworks for this are myriad [2-6]. Two key features of access-oriented studies are their focus on barriers to access, and their focus on attributes of individuals and communities. Findings of such studies are often that financial, educational and cultural issues need to be addressed, with conclusions drawn that surmounting such barriers will enable universal uptake of services.

In the case of access to health care for malaria, surveys have shown that treatment and even diagnostics are often procured outside of public sector services. Febrile patients are treated in many arenas, from the home, to drug shops, to private clinics and public health facilities. The majority of treatment for malaria is sought outside of the home and outside of the public sector in Africa [7]. This begs the question that services designed from the supply side, as in the case of public services, may not meet demand. In spite of the huge amount of research into malaria treatment-seeking, gaps remain in our understanding of provider characteristics [8]. The focus on individual and community 'barriers' to access of public health services [9] overshadows important lessons that could be learned from decisions to access other health care services.

This paper reviews the literature to understand treatment-seeking beyond barriers to access, focusing on research that has described what clients are looking for in the providers they approach for their health care needs. Thus, it moves away from individual and household decision-making, and individual characteristics of population groups, to look at the characteristics of providers that attract clients. The review aims to enable public health services to learn what they are perceived to do well, and what other competitors in the health services marketplace are also doing well to attract clients.

## Methods

The keywords 'malaria' and 'treatment seek*' or 'health seek*' and 'Africa' were searched for in the following databases: Web of Science, IBSS and Medline. The citation lists of the papers meeting eligibility criteria were then read in order to identify further papers, books, reports or other grey literature considered potentially relevant to the topic from the title. Abstracts were reviewed for each paper considered potentially relevant from its title, and those that remained potentially relevant were then retrieved and reviewed in full. The eligibility criteria were as follows:

Inclusion criteria

• About treatment-seeking for illnesses including malaria in Africa

• Describes provider characteristics found to positively affect patient access or choice to use that provider

• Presents analysis of primary empirical data

• Published any date before April 2011

• Does not meet exclusion criteria

Exclusion criteria

• Papers only focused on self-treatment

• Papers only focused on patient/household characteristics associated with provider use

• Data collected outside Africa

• Review or opinion papers without new data presented

• Papers in languages other than English

• Papers for which the full text could not be found, including conference presentations

• PhD theses

Reviews of each paper were undertaken by two members of the team. After reading the whole paper, reviewers extracted specific information into an Excel spreadsheet, detailing the location, study population and methods of the study as well as rationales given for preferred treatment sources in that population. The different rationales given across the different studies were then reviewed and a list of categories of 'attracting factors' was generated by grouping thematically all provider factors reported to have had a positive effect on decisions to seek treatment at that provider. A matrix was then constructed, such that papers could be entered according to the factor presented and the type of provider this was relevant to, where specified. For each paper, strength of evidence was assessed by evaluating (1) the methods used that contributed to the findings included in the review matrix, with open-ended methods considered more useful for eliciting relevant information than closed-ended methods, and (2) the richness of descriptions of findings relevant to the review's objective.

The papers and categories of attracting factors emerging were also reviewed by publication date, to assess any changes in factors reported over time. Papers were grouped by decade and the matrix of attracting factors and provider types was reviewed for contributions from papers from each decade.

## Results

### Papers identified and included

After screening titles, the sources searched provided the following number of potentially relevant papers/books (hereafter referred to as 'papers'):

- Web of Science: 157

- IBSS: 20

- Medline: 69

- Reference lists of retrieved papers: 82

After removing duplicates, a total of 267 abstracts were reviewed. Neither abstract nor full text could be obtained for four references. A total of 114 references were excluded after reading the abstract. All remaining papers were read in full, except for five, which could not be obtained. Of these remaining 145 papers, 48 were excluded, leaving 97 papers that fulfilled the inclusion criteria.

The articles were published between the years 1986 and 2011, with a median year of publication of 2006, showing a rapid increase in studies published in the past few years.

### Populations of included studies

The studies included in the review were carried out in 14 different countries, with the majority conducted in East Africa: Tanzania (n = 26), Uganda (n = 15) and Kenya (n = 8) followed by West Africa, particularly in Nigeria (n = 18) and Ghana (7). Studies included from Francophone West Africa were few, in Burkina Faso (n = 3), Gambia (n = 2) and Gabon (n = 1). Between one and three papers provided data from each Malawi, Zambia, Mozambique and South Africa. Two North African countries were represented: Ethiopia (n = 7) and Sudan (n = 2). There were no studies eligible for inclusion from Central African countries.

Of the 97 papers included in this review, almost half, 48.5% (n = 47) focused on health-seeking for children, 46.4% (n = 45) focused on health-seeking for both children and adults, and 4.7% (n = 5) had a sole focus on adults. In all, 73.2% (n = 71) took place amongst rural populations, 20.6% (n = 20) were conducted in mixed rural/urban settings, and 6.2% (n = 6) were limited to urban areas.

### Methodologies of included studies

Methods used by studies to obtain information that is included in this review were qualitative and quantitative, and often mixed. Most commonly, fieldwork included in-depth or 'key informant' interviews (n = 45), survey questionnaires (n = 44) and focus group discussions (in 43 studies). Many also described using 'semi-structured interviews' (n = 18), although it is possible these interviews varied in their level of depth as description of questions was often vague. Ethnography or participant observation was carried out in 13 studies. Narratives, case histories and informal conversations were grouped under the category of in-depth/key informant interviews.

In all, 70% (n = 68) papers were classified as contributing 'stronger evidence' to the review, defined by the methods used and richness of descriptions of the findings. All of these papers had used methods that included on open-ended questioning, enabling respondent to give responses beyond pre-defined *a priori *categories (70%, n = 68). A total of 31% (n = 30) papers were also classified as providing rich descriptions of findings relevant to the review's objective.

### Factors reported to attract patients to providers

The findings of the review, shown as a matrix of attracting factors by provider type, can be seen in Table 1, 2, 3, 4, 5. The papers identified a number of factors positively affecting patient choice to attend different providers, 'attracting factors'. Higher-level providers included hospitals (reported in 27 publications), public health centres or dispensaries (n = 33) and private clinics or health centres (n = 23). Lower-level providers included pharmacies and drug shops (n = 33), traditional healers and local herbalists (n = 31) and community health workers (n = 11). Some factors attracting patients were reported to be important across the different provider types, whilst others were specific to different types of provider, often clustered into 'high level' and 'low level' providers.

**Table 1:** Matrix of factors reported by papers to attract care seekers to different provider types: Cost of services

*Provider type*	*Community health workers* *(n = 4)*	*Drug shop/pharmacy* *(n = 19)*	*Traditional healer* *(n = 4)*	*Private health centre/facility* *(n = 3)*	*Public health centre/facility* *(n = 10)*	*Hospital* *(n = 3)*	*Provider type not specified* *(n = 13)*
*Attracting factor*							
***Cost of services at the provider (including consultation, treatment, supplies)*** ***(n = 44)***	'Cheap' Ethiopia[10], Uganda[11](traditional birth attendants)	'Cheap' **Kenya**[14], Tanzania[15], Uganda [16-18] Nigeria [19], [20] Ghana [21], Ethiopia [22], Tanzania[23], Malawi[24], Nigeria [25,26]	'Cheap'**Burkina Faso**[33]	'Cheap' Uganda [16]	Free services Malawi [36]South Africa [37] Kenya [38], Tanzania [31] Ethiopia [39]	'Free services' **Kenya**[35]	'Free or cheap drugs' Uganda [45,46] Tanzania [47]
	'No charge' Uganda [12,13]	'Affordable' drug vendors Nigeria[27,28]Ghana[29]	'For those who find it difficult to pay' Tanzania [23], **Uganda**[32]	'Reduced cost' **Kenya**[35]	'Free if unable to pay' **Tanzania**[34]	'Low cost' Uganda[41]	'Inexpensive services' Nigeria [48] Ghana [21], **Nigeria**[20], **Kenya**[14], [38,49,50], Mozambique[51]Ethiopia [52]
		'Charge less than others' Kenya [30]	'Flexible in payment' **Tanzania**[34]	'Can get treatment on credit' Uganda [16], Nigeria [28]	'Free if Under 5yr olds'Gambia[40]	'Economical for finding out the cause of an illness' **Tanzania**[44]	Affordable **Tanzania**[53]
		'Can get treatment on credit' Uganda [16], Tanzania [31], Nigeria [27,19,26]		'Pay according to the treatment you get' **Kenya**[35]	'Low cost' Mozambique [37]Uganda[41], Sudan[42]		'Flexibility in mode of payment' **Kenya**[14]
		'Option of bargaining' **Nigeria**[20]		'Prices are negotiable' **Kenya**[35]	'Can get treatment on credit' Tanzania [31]		
		'Give medicine according to the money you can afford' **Uganda**[32]			'Covered by insurance' Ghana [43]		

Note: Papers providing a rich description of attracting factors are marked in bold; findings from methods involving open-ended questions are underlined.

**Table 2:** Matrix of factors reported by papers to attract care seekers to different provider types: Proximity and timeliness of services

*Provider type*	*Community health workers* *(n = 7)*	*Drug shop/pharmacy* *(n = 23)*	*Traditional healer* *(n = 5)*	*Private health centre/facility* *(n = 6)*	*Public health centre/facility* *(n = 10)*	*Hospital * *(n = 2)*	*Provider type not specified* *(n = 12)*
*Attracting factor*							
***Proximity or easy access/lower cost of transport (n = 42)***	'Close by' Uganda [54]Nigeria & Ghana & Uganda & Kenya[55], Ethiopia[10,13], Nigeria [56,57]	'Close by' Uganda [54,32,16,18], Ethiopia [22]Ghana[29], **Kenya**[14], Nigeria [28,26,58,57]**Malawi**[59,24]	'Close by' Uganda [16], Nigeria [20,58]	'Close by' Nigeria [28], Ghana [21]	'Close by' Health centre **Zambia**[60], Uganda[12,16], Gambia[40], Burkina Faso[61], Nigeria [62]	'Close by' Nigeria [58] Nigeria [58]Burkina Faso[61],	'Close by'Ethiopia [52]Sudan[64], Nigeria [20,48], Ghana [21] Tanzania [47,4]Uganda[46], Kenya [38]
	'Easy access' Uganda[17] (traditional birth attendants)	'Less travel' Ghana [43], Tanzania[15] Malawi [36]	'Easy access' **Uganda**[32]		'Easy access' Uganda[41]Kenya[63]		'Easy access' Mozambique[51], **Tanzania**[53], Kenya [49]
		'Save on transport cost' Nigeria [27] Ghana [21] Kenya [30]					Save on transport cost' Kenya [38], Mozambique[51], Ghana [21]

***Provider timeliness of services (opening hours and waiting times) (n = 21)***	'Short waiting time' Nigeria [56]	'Available 24 hrs' **Kenya**[14]**, Nigeria**[19]	'Available at convenient hours' **Tanzania **[34]	'Operate for longer hours' Ghana[65]	'Fast services for malaria cases' Nigeria [66]		'Short waiting time' Ethiopia [52], Ghana [21], Kenya [38,49]Uganda[46] Nigeria [48]
		'Longer opening hours' **Nigeria**[20]**, Uganda**[18]		'Short waiting time' **Kenya**[14], Nigeria [28]	'Short waiting time' Ethiopia [39]		
		'Convenient treatment times' Ghana [43]		'Provide faster services' **Kenya**[35]			
		'Short waiting time' Ghana [21,43], Ethiopia [22], Tanzania [31] Malawi [36], **Uganda**[18] **'Provide fast services' Uganda**[18]					
							

Note: Papers providing a rich description of attracting factors are marked in bold; findings from methods involving open-ended questions are underlined.

**Table 3:** Matrix of factors reported by papers to attract care seekers to different provider types: Belief in provider and ability to choose drugs

*Provider type*	*Community health workers* *(n = 3)*	*Drug shop/pharmacy* *(n = 7)*	*Traditional healer* *(n = 27)*	*Private health centre/facility* *(n = 10)*	*Public health centre/facility* *(n = 11)*	*Hospital * *(n = 8)*	*Provider type not specified* *(n = 8)*
*Attracting factor*							
***Belief the provider and/or their treatments can cure the illness (n = 51)***	'Their drugs work well' Nigeria & Ghana & Uganda & Kenya[55], Nigeria [57,56]	**Nigeria**[19,27], Uganda[17,18], Ghana[29]	Tanzania [34,67,44,69]Uganda[70], **Malawi**[59,24] Nigeria [20,71,19,26]**Kenya**[35], **Ghana**[72], **Uganda**[32], Burkina Faso[61], Gabon[73],	Ghana [82,21] Tanzania[83,84],Uganda[11,17]Nigeria[85,86]Kenya[35,63],	Ethiopia [87]**Zambia**[60], Nigeria [26,66,86], Burkina Faso[61], Gabon[73], **Malawi**[59], Tanzania [88], Uganda [89]	Gambia [90], **Nigeria**[85,20,86]**Tanzania **[44,76]Burkina Faso[61], Ghana [21]	**Tanzania **[68,67,91], Uganda[17,45] Burkina Faso [92], Ghana [21] Ethiopia [52]
			'Effective for convulsions associated to witchcraft' **Zambia**[60],		'Treatment is comprehensive' Kenya[14]		
			'Effective for convulsions' Nigeria [20,74], Tanzania [75,76]**Ethiopia**[77]				
			'Effective for illnesses with natural or spiritual cause' Zambia[78], Tanzania [79,80]Nigeria[74], Ghana [21]				
			'Effective for illness unusual in presentation' Nigeria [81],				

***Provider allows the patient to choose what they want (n = 2)***		'Can buy any quantity of drugs you want' Nigeria[26]					
		'Can make a choice on the kind of drug' Nigeria [58]					

Note: Papers providing a rich description of attracting factors are marked in bold; findings from methods involving open-ended questions are underlined.

**Table 4:** Matrix of factors reported by papers to attract care seekers to different provider types: Staff and service characteristics

*Provider type*	*Community health workers* *(n = 3)*	*Drug shop/pharmacy* *(n = 7)*	*Traditional healer* *(n = 2)*	*Private health centre/facility* *(n = 7)*	*Public health centre/facility* *(n = 8)*	*Hospital* *(n = 7)*	*Provider type not specified* *(n = 3)*
*Attracting factor*							
***Positive manner of staff*** ***(n = 10)***	'Show care to patients' Uganda[12] **Ethiopia**[13]	'Show care to patients' **Uganda **[18]	'Show care to patients' **Tanzania **[34], **Uganda**[32]	'Show care to patients' Uganda[70],	'Polite' Nigeria [62]	'Friendly attitude' Zambia[93]	'Build relationships with patients' Kenya [49]
	'Good communication' **Ethiopia**[13]	'Good communication' **Uganda**[18]		'Friendly attitude' Nigeria [28]		'Staff are dedicated' Zambia[93]	

***Qualification and experience of provider*** ***(n = 6)***					'For trained staff' Nigeria [66] Uganda [16]	'For skilled staff' Tanzania [47]	'For trained staff' **Nigeria**[20] **Zambia**[60]
					'For experienced staff' **Tanzania**[34], Uganda [16]		'Availability of a Doctor' **Nigeria**[20]

***Specialist services offered by provider*** ***(n = 17)***	'For Advice' **Ghana**[72]	'For Advice' **Ghana**[72], **Uganda**[32]	'Conduct home visits and provide follow-up care' **Uganda**[32]	'For advice' Tanzania [96]	'For First Aid' Ethiopia[95],	'For Advice' Kenya[100]	
		'For first Aid' Uganda [16], Ghana [94], Ethiopia[95], Malawi[24]		'For Advice and further examination' Tanzania [97]	'For injections' Tanzania [34]	'For testing /diagnosis' **Tanzania**[98]Nigeria[26]	
				'For first Aid' Uganda [16], Ethiopia[95]	'For blood transfusion' Tanzania[23]	'For thorough examination' Zambia[93]	
				'For testing' **Tanzania**[98]	'For admission' Tanzania [31]	'For admission Tanzania [47,31]	
					'For specialized care' Uganda[99]	'For surgery' Tanzania [47]	
						'For blood transfusion' Tanzania[23]	

Note: Papers providing a rich description of attracting factors are marked in bold; findings from methods involving open-ended questions are underlined.

**Table 5:** Matrix of factors reported by papers to attract care seekers to different provider types: Supplies and services seen as good or recommended

*Provider type*	*Community health workers* *(n = 3)*	*Drug shop/pharmacy* *(n = 10)*	*Traditional healer* *(n = 1)*	*Private health centre/facility* *(n = 8)*	*Public health centre/facility* *(n = 13)*	*Hospital* *(n = 11)*	*Provider type not specified* *(n = 10)*
*Attracting factor*							
***Provider supplies (mostly drugs) and facilities are good (n = 28)***	'Drug availability' Ethiopia [95]	**Tanzania **[91]		'Drug availability' **Kenya**[35]**Ethiopia**[13]	'Drug availability' **Zambia**[60]Uganda [102]	'Drug availability' Zambia[93,78]Uganda[41], Nigeria [25]	'Availability of drugs' Nigeria [20,48]Uganda[46], Ghana[21]
	'Staff availability' Nigeria & Ghana & Uganda & Kenya[55], **Nigeria**[57]	'Drug availability' Nigeria [28,26]**Tanzania**[53,101] Uganda [32], [102,18]**Ethiopia**[13]			'Well equipped' **Kenya**[49,38]**Tanzania**[34] Uganda [16]	'Drips available' **Tanzania**[101]	
		'Wide range of drugs' **Tanzania**[101] Malawi[24]			'Ward for in-patients' Tanzania [47]	'Diagnostic facilities' Zambia[93] **Tanzania**[101]	
					'Presence of laboratory' Tanzania [47]	'Staff availability' Zambia[93]	
					'Medical staff' Kenya [38]		

***Better 'quality of services' (as a general statement) (n = 15)***		**Uganda**[18]	**Uganda**[32]	**Kenya**[14], Ethiopia[10], Uganda[41], Ghana [21]	Ethiopia [39] Nigeria [62]	Uganda[41], Nigeria [62]	Kenya[50,49], Tanzania [47], Nigeria [20,48], Mozambique[51], Ghana [21]
					'Have better quality control and regulation of services' Nigeria [66]		

***Provider recommended by another provider (n = 11)***				**Tanzania **[91,83]	Uganda[12,103,17]	Tanzania [69,104,34,75] Uganda [103],	'Health facilities ' Tanzania [105] **Burkina Faso**[33]

Note: Papers providing a rich description of attracting factors are marked in bold; findings from methods involving open-ended questions are underlined

### Factors important across all provider types

Interestingly, many of the factors reported to attract patients to providers were important across the different provider types. These included low cost of services, proximity of treatment source to the patient, a belief that the provider or their medicines could cure the illness and positive manner of the provider.

Low cost of services encapsulated reports of preferences for, or improved access to, services that were low in absolute cost or for which patients could negotiate over price or quantities or receive on credit. The former attributes, especially free services, were associated with public facilities or local herbs whilst the latter attributes were most commonly identified with drug shops.

Proximity or easy access to the health care providers was also a key factor reported, reflecting preferences for providers located nearby or in a location to where transport was easy to secure and afford. Provider timeliness of services included convenient opening hours and short waiting times. Longer opening hours of drug shops appealed to patients requiring attention at night or at weekends.

Provider supplies that were reported to attract patients were most commonly the availability of drugs, although specific facilities were also attractive, particularly having a laboratory, a ward, and good infrastructure, equipment like microscopes, injections and drips, most commonly reported as attracting factors at public health facilities. These also related to the desire for specialist services, including investigations, transfusions, surgery and admissions at higher-level facilities, whilst first aid was cited as an attracting factor at lower-level providers, such as drug shops and community health workers.

Papers also reported that patients were attracted to providers where they believed the treatment or medicines would cure their illness. For example, many reported recognition of traditional healers as the best to treat cases of convulsions or illnesses with spiritual causes. Patients were reported to attend biomedical health facilities because they believed that these facilities give adequate and appropriate treatment that would cure their illnesses.

Positive attitudes by provider, including being friendly, polite, providing consolation and showing concern were important across all provider types. Health seekers were reported to desire the provision of health services with dignity, respect, and humility on the part of the provider. Many of the studies in this category used open-ended questions, a method classified as stronger evidence, although the absolute numbers were few. Several described important attitudes in health worker in terms of being friendly to patients [28,94], offering counselling and consolation [18,34] and being caring in general [12,70].

### Attracting factors to higher-level providers

Specific attractions to higher-level providers were the qualifications of staff, perceived better 'quality of services', and recommendations to attend from another provider.

Higher-level facilities were frequently reported to be attractive due to the perceived higher level of formal training and experience of providers. Patients were reported to perceive these providers as competent, with greater expertise than others, and best suited to treat symptoms associated with uncomplicated malaria.

Private health clinics/health centres, public health facilities and hospitals were reported to be visited for their better 'quality of services'. Unfortunately, in many cases, this phrase was used without further explanation or deconstruction, possibly reflecting the nature of the studies, which used closed-question methods. However, in some cases, the term 'quality of services' was explained to include laboratory services, availability of drugs and qualified personnel. Others referred it as prompt diagnosis, appropriate treatment and good follow-up advice [48].

Other factors attracting people to providers included following recommendations from another provider. Such recommendations or referral, were described in cases where the initial provider was unable to treat the illness, when they would recommend another provider who was believed to be able treat that illness, or in cases when the provider recommended a facility where investigations or more complicated procedures such as blood transfusions could be carried out.

### Attracting factors to lower-level providers

Specific factors reported to attract patients to lower-level providers were the timeliness of services, including convenience of opening hours and short waiting times, and the ability to have choice over what medicines to purchase.

Timeliness was reported as crucial in clients' choice of providers, and lower-level providers were therefore attractive because they operate for longer hours as well as at weekends, unlike public health facilities. They were also reported to be popular for the shorter waiting times. Patients were reported to appreciate that such timely services were possible because of the profit motive of drug shops and pharmacies.

Choice over medicines was only reported by a few studies, which mentioned that patients are satisfied by the ability to purchase specific desired medicines and in the quantities or dosages that they were able to afford or wished to purchase at that time.

### Changes in attracting factors and provider types reported over time

The majority (n = 77, 79%) of papers eligible for inclusion in this review were published between the year 2000 and 2009, with only 10 papers published before this point, including just one since 1980 [49]. No strong trends in the types of attracting factors presented by papers from different years were identified, with most factors being mentioned by papers before and after the year 2000. However, it was noted that 'beliefs a provider can cure an illness', 'proximity or easy access' and 'timeliness of services' were factors reported slightly more frequently, proportional to the number of papers included in that time period, before the year 2000. Conversely, reports of the importance of 'specialist services' as attracting patients to providers emerged only after the year 2000, when reports of 'provider supplies and facilities' were also slightly more common amongst included publications.

Papers from across the years included all provider types, although papers with a focus on drug shops and community health workers were more common since the year 2000.

## Discussion

This review brings together papers that describe attributes of providers reported to attract patients to seek their health care services, specifically for malaria in Africa. An increasing number of papers have been published since the year 2000 that address this topic. The findings of this review add to those that have focused on attributes of patients and their communities, such as education level and socio-economic status, when trying to understand factors affecting access to health care. The review identified that several categories of factors that were reported to attract patients to providers were relevant across different provider types and different study settings: low cost of services, proximity of treatment source to the patient, a belief that the provider or their medicines could cure the illness and positive manner of the provider. Additional categories of factors were noted to attract patients to either higher- or lower-level providers: specific attractions to higher-level providers were the qualifications of staff, perceived better 'quality of services', and recommendations to attend from another provider; specific factors reported to attract patients to lower-level providers were the timeliness of services, including convenience of opening hours and short waiting times, and the ability to have choice over what medicines to purchase. Each of the factors identified can be considered in the design of interventions to increase access to health care for malaria in African settings.

This review focused on providers offering care in cases of malaria. The findings echo those found in studies and reviews of factors attracting patients to providers for other health services, such as for maternal health care and other childhood illnesses. Similar categories can be identified amongst papers describing provider attributes that attracted patients for other health services, including costs, proximity, timeliness and supplies [106-116], and the importance of health worker personalities and relationships with patients were even more prominently cited [62,107,109,111,113,116-118]. The findings presented in this paper were drawn from 14 different countries across Africa, without stark differences by country, suggesting some generalization of findings. However, the papers reflect the countries from which much malaria research emanates, with no contributions found from Central Africa, for example, which limits generalization. The increase in numbers of papers since the year 2000 is notable, although the review also identified that many factors identified in earlier papers were replicated in later publications, with the exception of more emphasis in later papers on specialist services and supplies. This could reflect changes in qualities desired in providers or a change to the framing of the research questions by investigators, perhaps suggesting a trend towards understanding the role of commodities in health care.

A limitation of the interpretation of the review's findings as identifying those factors most important to patients is the methodology used in the individual studies. Many of the studies were surveys, in which potentially important factors are pre-defined. The studies that used more open-ended methodologies tended to echo some of the same findings as the closed-question surveys but they also picked up other issues, including the importance of a belief that a particular provider could cure an illness and of the nature of the provider's interactions with patients. This points to the need for studies to continue to take open-ended approaches, in order to allow unanticipated issues to emerge. It also suggests that the list of factors attracting patients to providers may not be exhaustive, and indeed may appear skewed towards certain factors because of the questions asked. In addition, it was hard to interpret some of the broader factors listed but not deconstructed by authors, such as 'quality', 'costs' and 'access.' Such concepts tend to be composed of several notions, and carry different meanings for different individuals and groups. The more in-depth studies provided more insights for the research question by untangling these concepts rather than assuming a shared meaning by participants, researchers and readers.

While the review did find 97 papers mentioning provider characteristics affecting patient choice, these were often mentioned secondarily to individual patient or community factors. Rich descriptions of provider characteristics that attracted patients were relatively few. This may reflect the dominance of the discourse in public health currently that conceptualizes the responsibility for health at the individual level, with interventions targeted at empowering individuals to do 'the right thing' through provision of education and removal of financial and cultural barriers [119]. In global health, this can be seen to have arisen as part of the rapid reversal of the priorities set out in the comprehensive primary health care approach taken in the Alma Ata Declaration of 1978. When this horizontal strategy was superseded by more targeted, vertical programmes that were argued to achieve greater gains more quickly [120], conceptualizations of local populations shifted from active partners of demand-driven services to passive recipients of supply-driven services [121]. The concept of access can be seen as coupled with the top-down delivery of services, exemplified by initiatives to increase access to medicines, immunizations, sanitation and other health commodities. The Millennium Development Goals (MDGs) crystallized this targeted approach to meeting population needs for health care, and the rhetoric to 'increase access' can be found in the detail of all of the health-related MDGs [122].

Learning lessons from the literature on factors attracting patients to providers should include a reflection by policy makers and programme implementers about the assumptions made in the provision of services. Are patients expected to be responsible 'citizens', eager to seek care at 'appropriate' health facilities, as long as they have been educated and empowered financially? Or should patients be considered as agents, weighing up multiple desires and constraints, including the appeal of one provider over another? This literature review suggests that traditional barriers such as cost and proximity are important in access to health care. However, it also suggests that health services may increase their appeal if they were more responsive to client demands in terms of friendliness, timeliness, compassion and effectiveness. Multidimensional frameworks of health care access have described it as a degree of 'fit' between health care systems and individuals, households and communities [123]. This paper suggests that health services be proactive in developing their characteristics in line with patient preferences in order to evolve a good 'fit' with patient if they intend to improve health outcomes through access.

## Conclusions

Concerns over how to improve access to health services have tended to focus on characteristics of patients and their communities. This paper has reviewed characteristics about providers that attract patients to attend. Several characteristics are presented that were reported to attract patients to providers of all types, including lower cost of services, close proximity to patients, positive manner of providers, medicines that patients believe will cure them, and timeliness of services. The paper argues that improving access to services requires attention to those factors that will attract patients, and recommends that public services are improved in the specific aspects identified in this review. The paper also argues that research should expand its lens to understand provider characteristics more carefully, especially using methods that enable open responses. Access must be reconceptualized beyond the notion of barriers to consider attributes of attraction if patients are to receive quality care quickly.

## Competing interests

The authors declare that they have no competing interests.

## Authors' contributions

The review was conceived and designed by CIRC and SGS, papers were retrieved and analysed by JK, MK and CN, all authors contributed to drafting the manuscript and gave final approval of the version to be published.
